# Comprehensive Spatial Investigation of Tuberculosis Dynamics and Affecting Factors in Şanlıurfa, Türkiye (2016–2023)

**DOI:** 10.1029/2024GH001235

**Published:** 2025-07-22

**Authors:** M. Çelik, M. F. Döker, C. Kırlangıçoğlu, Ö. Ünsal, S. Gökçeoğlu, M. R. Ceylan, O. Karabay

**Affiliations:** ^1^ Faculty of Medicine Department of Infectious Diseases and Clinical Microbiology University of Harran Şanlıurfa Türkiye; ^2^ Faculty of Humanities and Social Sciences Department of Geography University of Sakarya Sakarya Türkiye; ^3^ Faculty of Art Design and Architecture Department of Architecture University of Sakarya Sakarya Türkiye; ^4^ Faculty of Arts and Sciences Department of Geography Bursa Uludağ University Bursa Türkiye; ^5^ Şanlıurfa Provincial Health Directorate Şanlıurfa Türkiye; ^6^ Faculty of Medicine Department of Infectious Diseases and Clinical Microbiology University of Harran Sanliurfa Turkey; ^7^ Faculty of Medicine Department of Infectious Diseases and Clinical Microbiology University of Sakarya Sakarya Türkiye

**Keywords:** tuberculosis, spatial analysis, geographic information systems, epidemiology, public health, socio‐economic factors

## Abstract

Tuberculosis (TB) remains a critical public health issue, particularly in regions with significant socio‐economic disparities. This study provides a comprehensive spatial analysis of TB dynamics in Şanlıurfa, Türkiye, covering the period from 2016 to 2023. Utilizing Geographic Information Systems, epidemiological data, and advanced statistical techniques, the research examines the spatial distribution and temporal trends of TB cases within this region. By integrating patient data with demographic, environmental, and socio‐economic variables, the study assesses the complex factors influencing TB incidence and prevalence. The results indicate significant spatial clustering of TB cases, with the highest concentrations in areas characterized by high population density, lower socio‐economic status, limited healthcare accessibility, and poor environmental conditions. Temporal trends reveal a gradual decline in TB incidence over the study period; however, certain hotspots persist, underscoring the need for sustained and targeted interventions. Furthermore, the study identifies a correlation between TB prevalence and inadequate living conditions, emphasizing the role of socio‐economic improvement in disease control. These findings provide crucial insights for policymakers and public health officials, facilitating the development of more effective, evidence‐based TB control strategies tailored to the unique socio‐economic and geographical landscape of Şanlıurfa.

## Key Points

- Tuberculosis cases cluster in Şanlıurfa's peripheral areas, linked to low socio‐economic status and limited healthcare access
- Tuberculosis incidence correlates with air pollution (PM 2.5) and respiratory infections, with urban density as a key factor
- Targeted health interventions, pollution control, and socio‐economic support are crucial for Tuberculosis control in high‐risk areas

## 1 Introduction

Tuberculosis (TB) is an infectious disease caused by bacteria belonging to the Mycobacterium tuberculosis complex. TB is one of the oldest known diseases throughout history and remains a significant cause of mortality. TB continues to represent a significant public health concern in the present era (Natarajan et al., 2020). As indicated in the Global TB Report, 7.5 million individuals were diagnosed with TB in 2022, with an estimated 1.3 million of them succumbing to the disease (WHO, 2024). While the M. tuberculosis complex can be transmitted via means such as the consumption of unpasteurized milk and direct inoculation, the most common transmission route is via the airway (Yates et al., 2016). The organs most commonly affected by TB are the lungs. However, M. tuberculosis can also spread to other body parts via the lymphohematogenous route, resulting in the development of extrapulmonary TB (Loddenkemper et al., 2016). TB has historically been associated with poverty. The majority of individuals who succumb to the disease annually reside in developing countries. Nevertheless, cases of tuberculosis have also been documented among economically disadvantaged populations in developed countries. For centuries, there have been anecdotal reports associating TB with environmental risk factors linked to poverty, including indoor air pollution, tobacco smoke, malnutrition, overcrowded living conditions, and excessive alcohol consumption (Schmidt, 2008).

While the impact of environmental factors is generally more limited in non‐communicable diseases, some infectious diseases, such as TB and the human immunodeficiency virus, demonstrate an extremely strong association with environmental factors. The rapid urbanization and industrialization processes in Europe, particularly during the Industrial Revolution, played a critical role in the geographical spread of TB to much larger areas. During this period, the proliferation of TB was exacerbated by a confluence of factors, including poor living conditions, malnutrition, overcrowding, inadequate infrastructure, and unhygienic environments, particularly prevalent in impoverished working‐class neighborhoods within major cities. The social and environmental changes brought about by the Industrial Revolution played a pivotal role in the spread of the disease, contributing to its emergence as one of the primary causes of mortality in Europe during this period (Barberis et al., 2017). The development and implementation of effective social and health policies to reduce the risk factors associated with the disease have been significantly influenced by the various definitions of the disease.

A review of the geographical distribution of TB diagnoses reveals that the highest infection rates are observed in Southeast Asia (46%), Africa (23%), and the Western Pacific (18%). Lower rates were documented in the Eastern Mediterranean (8.1%), the Americas (3.1%), and Europe (2.2%) (WHO, 2024). Türkiye is among the regions where the incidence of TB is relatively low, with an incidence of 10–49 per 100,000 people in 2021 (WHO, 2024). The decrease in the number of TB cases observed in Türkiye over the past decade can be predominantly attributed to the implementation of mobile screening initiatives and systematic case follow‐up management in designated health facilities (Ministry of Health of Türkiye, 2024). By examining the spatial distribution of environmental risk factors that have been demonstrated to influence the transmission of the disease and incorporating the findings into prevention strategies, it is anticipated that the objective of curbing the TB epidemic by 2030 as outlined in the UN's Sustainable Development Goals (SDG No: 3.3) in the health sector can be accomplished in Türkiye. Thus, the present study concentrates on TB‐specific geographical epidemiology, encompassing the examination of the spatial distribution of morbidity and mortality rates of the disease, and the elucidation of the relationship between this distribution and environmental, social, and economic factors. This approach furnishes pivotal information for disease control and prevention by analyzing climatic and environmental conditions that influence the spread of TB. A substantial corpus of research has demonstrated that the geographical distribution of TB is particularly concentrated in certain regions, and that this concentration is strongly associated with environmental factors. For instance, a study by Beiranvand et al. (2016) examined the relationship between climate and geographic distribution of TB using geographic information systems (GIS). This study underscores the significance of comprehending the spatial distribution of TB as a critical evidence‐based instrument for disease control and prevention. TB‐specific geographic epidemiology facilitates a more profound comprehension of the disease within environmental and socioeconomic contexts, thereby contributing to the development of effective public health interventions (Ugwu et al., 2021).

GIS is an effective tool that is frequently utilized in epidemiologic studies, offering efficacious map‐based analytical opportunities to reveal the spatial distribution of epidemics (GUO et al., 2017). GIS‐based analyses have the potential to identify the geographical regions in which TB clusters are present, thereby providing healthcare providers with valuable information that can inform the planning, implementation, monitoring, and evaluation of TB control strategies (Alene et al., 2021). Spatio‐temporal analyses have previously been employed in a number of studies to investigate risk factors for malaria, schistosomiasis, and hand‐foot‐and‐mouth disease (Liao et al., 2016; Mabaso et al., 2006; Yang et al., 2005). Some studies on TB have demonstrated that cases cluster in specific locations and times, and that the distribution of TB is heterogeneous (Jinou et al., 2019; L. Li et al., 2016; T. Li et al., 2017; M.‐Y. Liu et al., 2018; Xia et al., 2020). A thorough examination of the extant studies (Yang Wenbai., 2019; Cui et al., 2019; Kiani et al., 2021; Wardani & Wahono, 2020; Dangisso et al., 2020; Fatima et al., 2024) that have investigated the correlation between TB cases and environmental factors reveals new analysis techniques related with spatial and temporal distribution statistics. These studies were predominantly conducted at the provincial, district, and neighborhood levels, primarily by researchers specializing in spatial epidemiology. In contrast, the present study employs a multidisciplinary approach, integrating spatiotemporal analyses (utilizing the space‐time cube method) (ESRI, 2024a) and environmental determinants of disease (employing the Multiscale Geographic Weighted Regression (MGWR) framework) in a simultaneous investigation. This study utilizes micro‐level data (anonymized patient locations and diagnosis times), distinguishing itself from previous studies. This methodological approach sets our study apart from previous research and offers a novel perspective on the relationship between environmental factors, disease occurrence, and patient location.

The study also aims to examine the relationship between TB and sociocultural and environmental factors using spatial analysis methods. The study employs a variety of data on patients diagnosed with TB in Şanlıurfa province, located in southeastern Türkiye. The paucity of data on the geographical distribution of TB in Türkiye, coupled with the critical role these data play in the development of health policies, underscores the importance of this research. It is anticipated that the findings of the analysis will furnish valuable insights into the geographical distribution of TB and inform the development of more efficacious strategies for disease control.

## 2 Study Area

**Figure 1:**
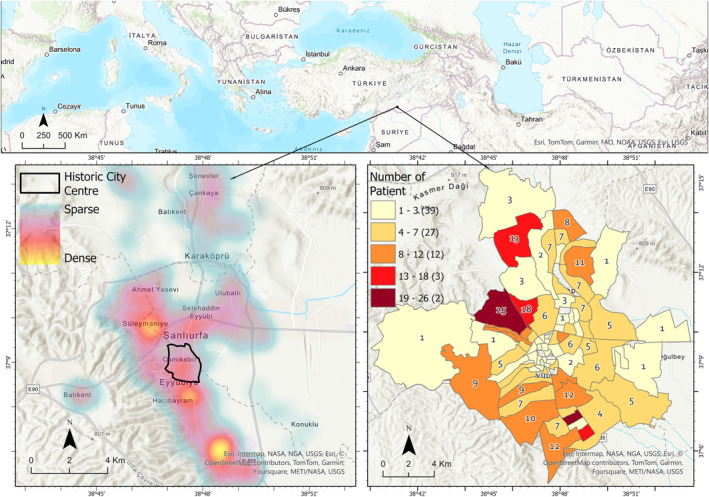
Location of the study area.

The study area comprises the central districts of Şanlıurfa Province which is among the most significant settlements in southeastern Türkiye (Figure 1), with a population of 2,213,964 and a surface area of 19,451 km^2^ with an average population density of 114 people per km^2^ (Republic of Türkiye Ministry of General Defence Directorate General For Mapping, 2024; Turkish Statistical Institute, 2023). In Figure 1, the bottom left shows the heat map of the patient locations used in the research, while the map on the bottom right shows the distribution of patients at the neighborhood level.

The city's strategic location at the core of the Southeastern Anatolia Regional Development Project, initiated in the 1990s, has elevated its significance. It has been inhabited since prehistoric times and has made a substantial contribution to its current status. The 223 km border with Syria to the south has positioned Şanlıurfa at the center of the political problems in the region, as a result of which the city has become a focal point for the region's political instability. Following the 2011 Syrian civil war, the influx of refugees into Türkiye had a direct impact on the demographic composition of Şanlıurfa. At the time of writing, 272,919 individuals of Syrian nationality are resident in the province, representing the third largest number of Syrians living in a Turkish city, after Istanbul and Gaziantep (Republic of Türkiye Ministry of Interior, 2024). Moreover, the city is home to migrants from a multitude of countries.

## 3 Material and Methods

**Figure 2:**
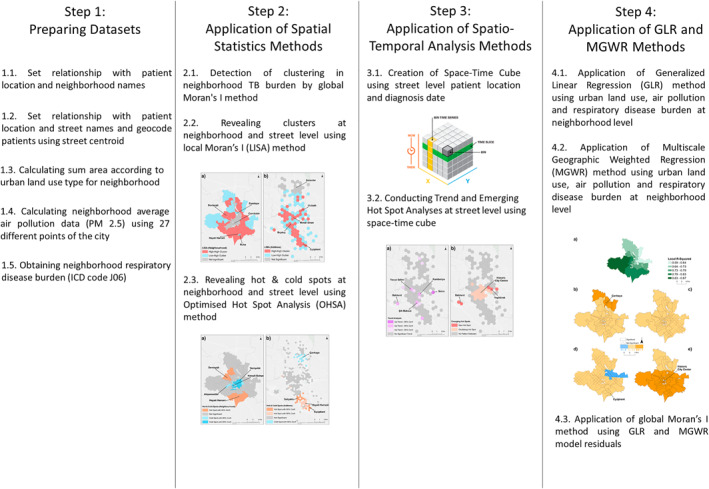
Workflow of the methodology.

The research was conducted in accordance with a predetermined methodology comprising ten discrete stages. The initial five stages entailed the preparation of data for analysis, while the subsequent five stages encompassed the application of analytical techniques (Figure 2). The process was conducted using MS Excel and ArcGIS Pro software.

### 3.1 Data Collection

The data and data sources utilized in the study are outlined below:

- Patient Data: Data (gender, age, nationality, time of diagnosis, address information) of patients diagnosed with TB between 2016 and 2023 were obtained from Şanlıurfa Central Tuberculosis Dispensary.
- Neighborhood and Street Data: Neighborhood boundaries and street data were obtained from Şanlıurfa Metropolitan Municipality.
- Urban Atlas Data: Urban land use classes produced within the scope of the Copernicus Land Monitoring Service project of the European Union were used (Copernicus Land Monitoring Service, 2024).
- Air Pollution and Respiratory Disease Data: PM 2.5 measurement data and respiratory disease data for 2021 were used (Vural & Şahinalp, 2022).

In the initial phase of the investigation, the geographical names associated with the patient data were linked to the corresponding neighborhood boundaries in the data set. This facilitated the mapping and analysis at the neighborhood level. In the 1.2 step of the research, the street names in the patient data were linked to the relevant street data. Subsequently, the center point (centroid) of the associated streets was generated, and the locations of the patients were transferred to the street‐level map. In the third stage, neighborhood‐level land use maps were produced from Urban Atlas data in Şanlıurfa. This process yielded data regarding the quantity and proportion of active green space within each neighborhood. In the fourth stage, neighborhood‐based air pollution maps were produced with the IDW interpolation method, whereby the average of PM 2.5 values measured in 27 different points of the city in 2021 was taken. In the fifth stage, data on total acute upper respiratory tract infections and multiple and unspecified locations (ICD code J06) in 2021 in tabular format and at the neighborhood level were associated with neighborhood boundaries.

### 3.2 Data Analysis

In the 2.1 stage, the spatial autocorrelation (Moran's I) was calculated using the number of patients in each neighborhood. This analysis was conducted to ascertain whether there was a clustering of cases at the neighborhood level. The Moran's I index is a crucial indicator for discerning the presence (positive or negative) and extent of spatial autocorrelation. In order to calculate the index value, an equation was produced by Patrick Alfred Pierce Moran (Moran, 1950).

(1) $$I=\frac{n}{S_{0}}\frac{\sum_{i=1}^{n}\sum_{j=1}^{n}w_{i,j}z_{i}z_{j}}{\sum_{i=1}^{n}z_{i}^{2}}$$

In this equation, *n* represents the total number of objects, zi represents the deviation from the mean value of the *i* th attribute, wi,j represents the spatial weight between the *i* th and *j* th values and S_0_ represents the sum of all weight values. The Moran's I index obtained from this analysis takes negative and positive values between −1 and +1. Positive values indicate that there is positive autocorrelation and similar values are spatially clustered; negative values indicate that there is negative autocorrelation and values are spatially dispersed. A value of zero (0) indicates the absence of spatial autocorrelation. In addition, the *Z* score and P value are calculated with this method. If the *Z* score is greater than 2.58 or less than −2.58 and the p‐value is less than 0.01, the result is attained with a confidence interval exceeding 99% (Getis & Ord, 1992; Goodchild MF, 1986; Griffith, 1987). The Global Moran's I method is employed to ascertain the presence of clustering in the data, while the Local Moran's I method is utilized to determine the location and nature of such clustering. In the present study, both global and local Moran's I methods were employed to analyze the geographical distribution of patients. The maps illustrating the clustering and outliers were meticulously prepared and interpreted. The Local Moran's I statistic (LISA) is a method used to demonstrate the occurrence of spatial clustering and the presence of outliers. In the 2.2 step, Anselin Local Moran's I analysis was conducted to elucidate the locations and mechanisms underlying the formation of clusters. A positive index value indicates the presence of clustering, whereas a negative value indicates the presence of spatial outliers. The LISA is calculated using two equations (Anselin, 1995).

(2) $$I_{i}=\frac{x_{i}-\bar{X}}{S_{i}^{2}}\sum_{J=1,j\neq i}^{n}w_{i,j}\left(x_{j}-\bar{X}\right)$$

(3) $$S_{i}^{2}=\frac{\sum_{j=1,j\neq i}^{n}{\left(x_{j}-\bar{X}\right)}^{2}}{n-1}$$

Equation 2, xi is the value of the *i*th object in the data set, X̅ is the mean of that value, wi,j represents the weight value between the *i*th and *j*th data points (26, 27). In Equation 3, *n* represents the total number of objects. In order to test the statistical significance of the data set in question, the Anselin Local Moran's I method employs the calculation of both a z‐score and a p‐value. This process allows for the identification of objects that are not statistically significant. The analysis yielded five distinct coding types, classified according to the observed clustering patterns and the presence of outlier values. These coding is as follows:

- High‐High Clustering (HH): The spatial entity with high value is surrounded by high values.
- Low‐Low Clustering (LL): Low‐value spatial assets are surrounded by low‐value spatial assets.
- High‐Low Outlier Value (HL): An object with a high attribute value is surrounded by elements with a low value.
- Low‐High Outlier (LH): An object with a low attribute value is surrounded by objects with a high value.
- Statistically Insignificant: Local Moran's I value close to zero.

LISA analysis was also performed using patient locations. This way, LISA findings at the neighborhood level and LISA findings generated using patient locations were compared. In the 2.3 step, an Optimized Hot Spot analysis was performed according to the number of patients at the neighborhood level. Hot spot analysis was developed by Art Getis and Keith Ord in 1995 (Ord & Getis, 1995). This analysis creates a statistically significant hot and cold spot map using the Getis‐Ord Gi* statistic in Equation 4.

(4) $$G_{i}^{*}=\frac{\sum_{j=1}^{n}w_{i,j\;}x_{j}-\bar{\bar{x}}\sum_{j=1}^{n}w_{i,j}}{\sqrt{\frac{\left[n\sum_{j-1}^{n}w_{i,j}^{2}-{\left(\sum_{j=1}^{n}w_{i,j}\right)}^{2}\right]}{n-1}}}$$

In Equation 4, xj is the value of the *j*th feature in the data set, wi,j is the spatial weight between the *i*th and *j*th data points, *n* is the total number of objects, and S is the standard deviation (23, 28). As the Gi* value calculated in the equation approaches zero, it is understood that there are no high or low values in the neighborhood of the calculated object. As the z‐score increases, it can be inferred that objects with high values in the geographic data are co‐located or clustered. This allows for the identification of hotspots. Conversely, a smaller z‐score indicates a greater likelihood of objects with low values being located together, leading to the emergence of cold spots. To achieve optimal results, it is recommended to employ optimized hotspot analysis in lieu of the classical hotspot analysis method. Optimized Hot Spot analysis generally reduces statistically insignificant results compared to Hot Spot Analysis, since it calculates the optimum distance band. Furthermore, this method determines the number of objects with fewer neighbors than expected, based on the distance band in meters provided for the geographical objects as input.

The hot and cold spot maps were generated through the implementation of an optimized hot spot analysis, with the number of patients at the neighborhood level serving as the primary variable. The maps comprise seven distinct classes, as determined by Gi* statistics (ESRI, 2024b). Negative values may assume the values −3, −2, or −1. Such areas are referred to as “cold spots.” An object with a value of −3 represents a cold spot at a 99% confidence level. In comparison, objects with a value of −2 represent cold spots at a 95% confidence level. An object with a value of −1 is defined as a cold spot with a confidence level of 90%. A value of 0 indicates that the object in question is not statistically significant. An object with a value of 1 is defined as a hotspot with a confidence level of 90%. Objects with a value of 2 are identified as hotspots with a 95% confidence level, while objects with a value of 3 are classified as hotspots with a 99% confidence level. Additionally, an optimized hot spot analysis was conducted using patient locations. This method entails a comparison of the optimized hot spot findings at the neighborhood level with those created through the analysis of patient locations. In the 3.1 step, spatio‐temporal analyses were conducted according to the time of diagnosis of the 458 patients identified on the map. To this end, a space‐time cube was initially constructed from the patient data. Subsequently, the upward and downward trends were identified through the implementation of the Mann‐Kendall trend analysis (Kendall, 1990; Mann, 1945). This test, which is an ordinal correlation method, is conducted for each location. The bin values of each slice in the space‐time cube are then compared. In all cases where the first value is less than the second, the result is +1; conversely, if the first value is greater, the result is −1. If the two values are equal, the result is 0. A weighted +1 result indicates a positive *z* score, and a *p*‐value less than 0.001 indicates an uptrend in that region at a 99% confidence level. A weighted negative result, a negative z‐score, and a *p*‐value less than 0.001 indicate a downward trend in that region at the 99% confidence level. A value of 0 indicates the absence of a temporal trend.

Subsequently, an Emerging Hot Spot analysis was conducted (ESRI, 2024b). The results are produced through the application of hot spot and Mann‐Kendall trend analysis, as previously outlined in the 2.3 step. In total, 17 distinct pattern types (including new hot spot, persistent hot spot, sporadic cold spot, and e.t.c.) can be identified. The findings of the 3.2 stage were presented in the form of automatically generated honeycombs, with the patient locations superimposed. In the 4.1 and 4.2 steps, the Generalized Linear Regression (GLR) and MGWR methods were employed to elucidate the distribution of tuberculosis patients by neighborhood in Şanlıurfa. The GLR method allows for the identification of the direction of the relationship between the dependent and independent variables, whether positive or negative.

(5) $$y_{i}=\beta_{0}+b_{1}x_{1\;}+b_{2}x_{2\;}+...b_{n}x_{n\;}+\varepsilon$$

where yi represents the dependent variable observation (number of tuberculosis patients living in the neighborhood) at the *i*th locations (neighbourhoods in Şanlıurfa city), β0 is the estimated intercept and indicates the value of *y* when *x* equals to zero, b1 is the parameter estimate for x1.xn denotes the set of explanatory variables. Also, bn represents the regression coefficients that describe changes in the dependent variable *y* when *x* changes by one unit. Prior to the application of GLR, all variables were standardized, and the model type was selected as continuous (Gaussian). The exploratory regression method was employed to ascertain the variable combination exhibiting the highest *R* ^2^ value within the GLR model. In this method, the minimum number of explanatory variables was four, the minimum acceptable adjusted *R* ^2^ value was 0.5, and the maximum VIF value was 7.5. Subsequently, Moran's I analysis was employed to ascertain whether there was a statistically significant clustering in the linear regression residuals. To address the clustering issue in the GLR model residuals, enhance the estimates, and gain deeper insights into regional heterogeneity, the MGWR approach was employed (step 4.3).

(6) $$y_{i}=\sum_{j=0}^{m}\beta_{bwj}\left(u_{i},v_{i}\right)x_{ij}+\varepsilon_{i}$$

Where γi is the response variable, βbwj is the bandwidth used in the *j*th location ((ui,vi)), xij is the *j*th predictor, and εi is the error term. This method employs a variable bandwidth and a distinct geographic weighting matrix for each geographic object. In comparison to GWR, which employs a fixed bandwidth and a differential weighting matrix, there is no reduction in the representation of correlations without simultaneously preventing both over‐ and under‐fitting of variables. These features have been shown to enhance model accuracy in numerous studies (Fotheringham et al., 2017, 2019; Senyel Kurkcuoglu, 2023; Sisman & Aydinoglu, 2022; Yang, 2014; Yilmaz & Ulubaş Hamurcu, 2022; Ünsal et al., 2023). Finally, as with the GLR residuals, we employed Moran's I to ascertain whether clustering was present in the MGWR residuals.

## 4 Results

### 4.1 Existence and Structure of Spatial Clustering

**Figure 3:**
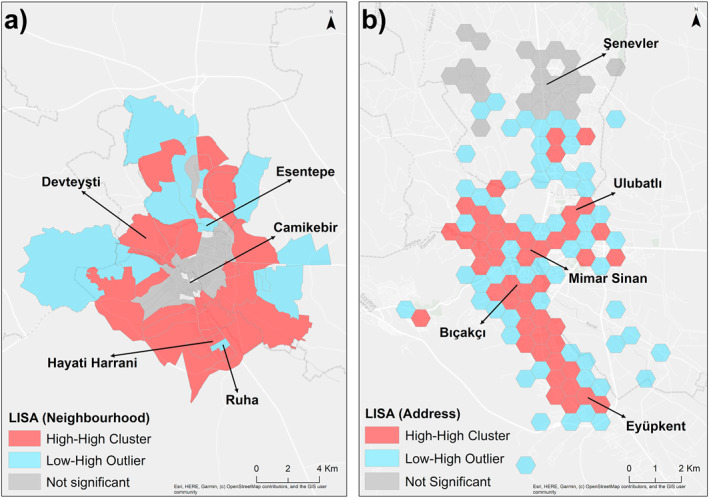
Spatial distribution of LISA: (a) Neighborhood; (b) Patient location.

The results of Moran's I analysis, conducted on the number of patients in 83 neighborhoods in Şanlıurfa, indicate a Moran's I value of 0.13, a z‐score of 5.28, and a *p*‐value of 0.000. The results demonstrate a statistically significant spatial clustering in the number of patients across neighborhoods. The results of the LISA analysis, conducted to ascertain the locations and mechanisms of clustering based on the number of patients in a given neighborhood, indicate the absence of statistically significant clusters or outlier groups in the neighborhoods within the historical city center. In contrast, the peripheral neighborhoods situated beyond the aforementioned center were classified as a High‐High Cluster. These neighborhoods constitute a cluster with other neighborhoods exhibiting a high number of patients. Low‐High Outlier neighborhoods indicated in blue on the map, exhibit a low number of patients and are situated in proximity to neighborhoods with a high number of patients (Figure 3a). The results indicate the formation of High‐High Cluster type honeycombs from the Bıçakçı and Pınarbaşı neighborhoods in the historical city center to the Eyüpkent neighborhood in the southeast. Additionally, High‐High Cluster type honeycombs are observed in the vicinity of Devteyşti, Yavuz Selim, Akabe, Direkli, Mimar Sinan, Şair Nabi, and Ulubatlı neighborhoods. Moreover, the LISA analysis yielded results indicating that honeycombs comprising a high number of patients were situated in close proximity to one another in these regions, particularly in relation to the location of patients and the time of diagnosis. In general, the honeycombs on the outer periphery of these regions are of the Low‐High Outlier type. Furthermore, the honeycombs that are not statistically significant are predominantly located in the northern neighborhoods of the city (Figure 3b).

**Figure 4:**
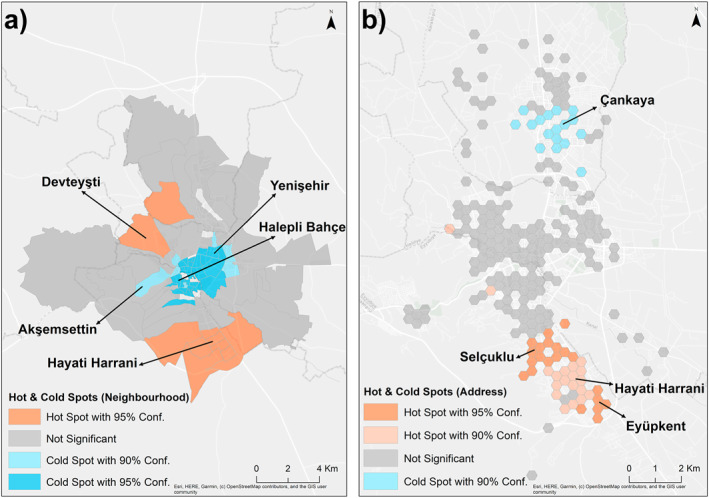
Spatial distribution of Hot and Cold Spot analysis: (a) Neighborhood; (b) Patient location.

The results of the Optimized Hot Spot analysis, based on the number of patients in each neighborhood, indicate the presence of hot spots at a 95% confidence level in eight neighborhoods in the southeast and two neighborhoods in the northwest. Conversely, the historic city center and the neighborhoods to the northeast of it have been identified as exhibiting cold spots at a 95% confidence level. In seven neighborhoods situated on the periphery of the aforementioned cluster, a cold spot was identified at the 90% confidence level. No statistically significant class was identified in any of the other neighborhoods (Figure 4a).

The results of the Optimized Hot Spot analysis, conducted using patient locations, identified the presence of hot spots in the southeastern region of the city at both the 90% and 95% confidence levels. The neighborhoods of Eyyüp Nebi, Selçuklu, and Eyüpkent exhibited hot spots at the 95% confidence level, while the neighborhoods of Hayati Harrani and Ruha demonstrated hot spots at the 90% confidence level. However, at the 90% confidence level, cold spots were identified in the neighborhoods of Doğukent, Çankaya, and Akpıyar in the northern part of the city. Furthermore, no statistically significant region was identified in the majority of the city (Figure 4b).

### 4.2 Spatiotemporal Pattern Analysis Results

**Figure 5:**
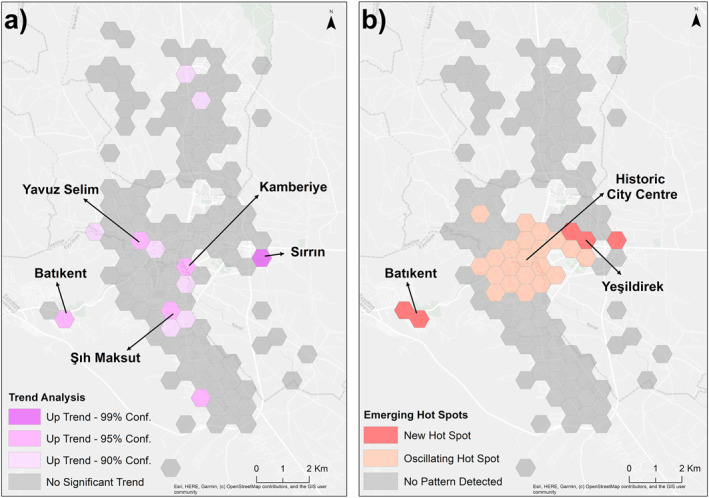
Spatial distribution of space‐time pattern mining results: (a) trend analysis; (b) emerging hot spot analysis.

The results of the space‐time pattern mining indicate an upward trend in the number of patients diagnosed at specific times and locations across the city. This trend was identified in 13 honeycombs. Of these, only one honeycomb is associated with the highest level of confidence (99%). The aforementioned honeycomb is situated in the Sırrın neighborhood, located to the southeast of the intersection of Recep Tayyip Erdoğan Boulevard and Şanlıurfa ring road. Honeycombs with 90% and 95% confidence levels were also identified in Batıkent, Yavuz Selim, Bağlarbaşı, Kamberiye, Kurtuluş, Şıh Maksut, and Muradiye neighborhoods. The upward‐trending honeycombs were found to be independently located in the Osmanlı, Batıkent, Süleymaniye, and Direkli neighborhoods. No statistically significant trend was identified in the remaining honeycombs (Figure 5a). Additionally, the emergence of new hotspots was observed in five distinct regions within the city. The aforementioned areas are situated within the confines of the Yenişehir, Yeşildirek, Karşıyaka, and Batıkent neighborhoods. Additionally, an oscillating hot spot was identified in the historical city center and its north‐northeast line. Moreover, a statistically significant hot spot was identified in this region during the most recent time interval, while a statistically significant cold spot was observed in the preceding time step. No statistically significant pattern is evident in the remaining honeycombs (Figure 5b).

### 4.3 Association of Disease Burden With Environmental Variables

The GLR model for the number of TB patients yielded an Akaike Information Criterion (AIC) value of 458.78 and an adjusted *R* ^2^ of 0.52, indicating an explanatory power of approximately 52% concerning the TB patients in the neighborhood within the study area. It is noteworthy that the results demonstrated statistical significance (*p* < 0.01) for the explanatory variables (Table 1). Furthermore, the Jarque–Bera Statistic registered at 129.10, the Koenker (BP) Statistic at 9.15, the Joint F‐Statistic at 24.25, and the Joint Wald Statistic at 65.77. The aforementioned values indicate that the GLR residuals exhibit a normal distribution. The GLR findings indicate a statistically significant positive association between the incidence of acute upper respiratory infections of multiple and unspecified sites and the number of tuberculosis patients in urban neighborhoods of Sanliurfa (Table 1).

**Table 1:** Summary of GLR Results

Variable	Coeff.	StdErr	t‐Statistic	Prob.	RobustSE	Robust_t	Robust_Pr
Intercept	−3.598	2.18	−1.64	0.10	2.56	−1.40	0.16
Continuous urban fabric	0.000018	0.00	5.31	0.00	0.00	6.06	0.00*
Roads and associated land	0.000005	0.00	2.73	0.00	0.00	2.48	0.01*
PM 2.5 average	0.175	0.07	2.24	0.02	0.09	1.81	0.01*
Acute upper respiratory infections	0.002	0.00	4.97	0.00	0.00	4.10	0.00*

The Moran's Index was employed to guarantee that the residuals of the GLR model are randomly distributed in accordance with spatial principles. The residuals of the GLR model were found to exhibit a random distribution at the neighborhood level, with a Moran's Index value of 0.008. This finding is not statistically significant, with a z‐score of 0.67 and a *p*‐value of 0.50. As a non‐spatial regression model, GLR demonstrated the direction of the relationships. However, MGWR contributes to estimating the local relationship between the number of TB patients in urban neighborhoods of Şanlıurfa and other variables, as well as addressing the issues of non‐stationarity and heteroscedasticity. The adjusted *R* ^2^ values for the number of TB patients in urban neighborhoods of Şanlıurfa obtained with the MGWR model were superior to those obtained with the GLR model. The MGWR model yielded an adjusted *R* ^2^ value of 0.75 for the number of TB patients in urban neighborhoods of Şanlıurfa. Moreover, the AICc was recorded at 145.19, the Sigma‐Squared at 0.24, the Sigma‐Squared MLE at 0.20, and the Effective Degrees of Freedom at 71.83. The MGWR bandwidth range for the number of TB patients in urban neighborhoods of Şanlıurfa is between 12 and 86 (Table 2).

**Table 2:** Summary of MGWR Results

Explanatory variables	Neighbors (% of Features)	Significance (% of Features)
Intercept	60 (69.77)	19 (22.09)
Continuous urban fabric	78 (90.7)	86 (100)
Roads and associated land	81 (94.19)	68 (79.07)
PM 2.5 average	51 (59.3)	12 (13.95)
Acute upper respiratory infections	48 (55.81)	86 (100)

**Figure 6:**
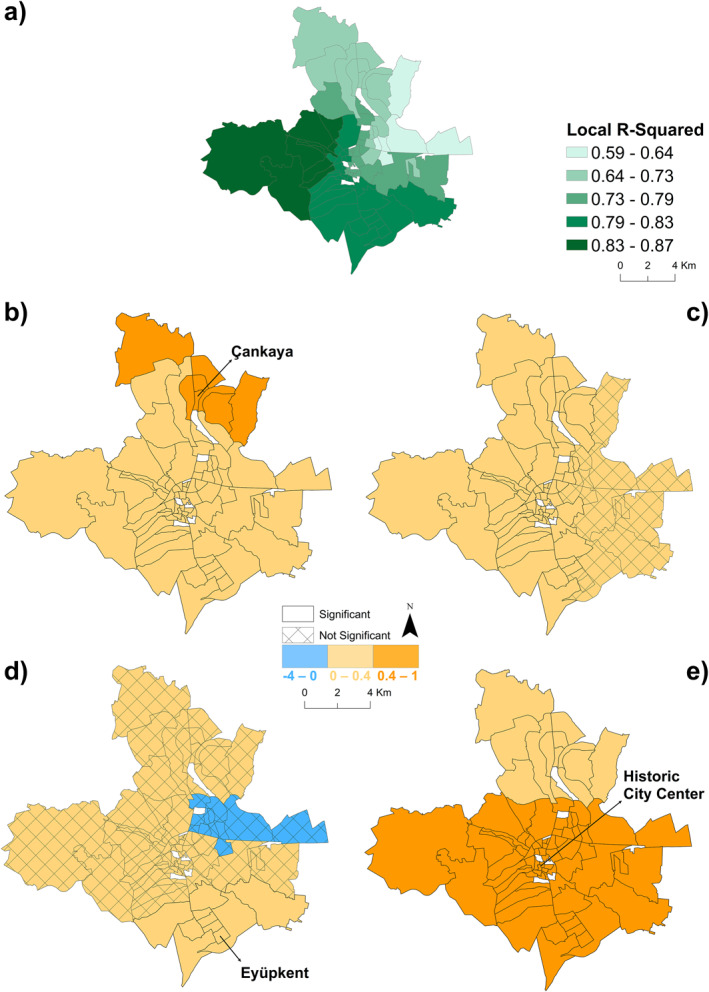
(a) Spatial distribution of multiscale geographic weighted regression (MGWR) local *R* ^2^ values; (b) Spatial distribution of MGWR model coefficients and significance status: Continuous Urban Fabric (S.L. >80%); (c) Roads and Associated Land; (d) PM 2.5 Average; (e) Acute upper respiratory infections.

The Moran's I test was also applied to the MGWR model. Accordingly, the Moran's I value of the scaled standardized residuals in the number of TB patients MGWR model was −0.02, with a *p*‐value of 0.65 and a z‐score of −0.44. These values indicate that the residual values in the number of TB patients in urban neighborhoods of Şanlıurfa are not statistically significant. The MGWR model developed in this study was employed to assess the degree of fit of the selected explanatory variables on the number of TB patients in each urban neighborhood of Şanlıurfa (Figure 6a). The results demonstrated that the local R^2^ goodness of fit effect was favorable in the majority of Şanlıurfa's urban neighborhoods. A goodness of fit exceeding 0.75 was observed in 69.7% of the urban neighborhoods of Şanlıurfa. It has been demonstrated that the selected impact factors exhibit enhanced explanatory power when moving from east to west. The correlation between the number of TB patients in urban neighborhoods of Şanlıurfa and the proportion of urban fabric (S. L. >80%) is positive and statistically significant in all neighborhoods. This relationship is further reinforced in 12 neighborhoods in the northern region (Figure 6b). The correlation between the number of tuberculosis (TB) patients in urban neighborhoods of Şanlıurfa and the presence of rapid transit and other roads and the associated land is positive and predominantly (81.9%) statistically significant. In accordance with the model's accuracy, some neighborhoods are not statistically significant in the eastern neighborhoods, where the accuracy is lower (Figure 6c). The correlation between the prevalence of tuberculosis (TB) patients in urban neighborhoods of Şanlıurfa and the concentration of fine particulate matter (PM 2.5) during the autumn of 2021 is statistically significant and positive in only 10 neighborhoods situated in the southeastern region of the city. This correlation is particularly pronounced in the Eyüpkent, Asya, and Konuklu neighborhoods (Figure 6d). The correlation between the number of TB patients in urban neighborhoods of Şanlıurfa and the incidence of acute upper respiratory infections of multiple and unspecified sites (sum of 2021) is positive and statistically significant in all neighborhoods. This relationship is observed to intensify in a south‐to‐north direction. The correlation is particularly strong in the historic city center and 33 neighborhoods in the south‐southeast (Figure 6e).

## 5 Discussion

The study's objective was to examine the correlation between socio‐cultural and environmental factors in patients diagnosed with TB in Şanlıurfa province, which is located in southeastern Türkiye. Utilizing spatial analyses, the study identified that cases of tuberculosis are concentrated in specific neighborhoods with high levels of particulate matter (PM 2.5). Furthermore, the socio‐economic levels of neighborhoods with a high concentration of tuberculosis cases are lower. Furthermore, a statistically significant correlation and spatial clustering were identified between the number of patients diagnosed with an acute upper respiratory tract infection and the number of patients diagnosed with TB in specific regions.

The utilization of GIS in the collection of spatial epidemiologic data has the potential to facilitate the monitoring and prevention of diseases, the management of outbreaks, and the provision of research‐based medical facilities (I. Fatima et al., 2021). In a study conducted in the South Punjab province of Pakistan by M. Fatima et al. (2024), it was found that 26.8% of TB cases were clustered in the central part of the region. In Brazil, De Abreu E Silva et al. (2016) identified a spatial clustering of TB cases in urban areas with high population density, relative to the rest of the city. The authors concluded that population density was a significant factor associated with the incidence of TB cases. A study conducted in Beijing, China demonstrated that TB cases exhibited spatio‐temporal clustering patterns contingent on population density, mobility, and socio‐economic status (Y. Liu et al., 2012). Beiranvand et al. (2016) evaluated the effect of climate and average annual rainfall rate on TB incidence in Iran using GIS and reported that the cumulative incidence of TB was high in extremely arid areas and that there was a significant inverse correlation between annual rainfall and TB incidence rate. In Pakistan, Shaikh and Malik (Shaikh & Malik, 2019) employed Local Moran's I clustering techniques to identify tuberculosis hotspots and cold spots at the country level. In this study, the Global Moran's I value was determined to be 0.25238, and the permutation test yielded a *p*‐value of 0.001. In a study conducted in China by Alene et al. (2021), the spatial clustering of TB was determined using Moran's I and Getis‐Ord analyses. The results indicated the presence of hot spots in the western part of Hunan Province, as well as spatial clustering of TB incidence at the provincial level.

This study employed Moran's I to determine the presence of any potential clustering of cases at the neighborhood level. To elucidate the nature and extent of the observed clustering, LISA was utilized. The identification of “hot spots” was facilitated by employing the Optimized Hot Spot method, which utilizes patient locations to determine spatial patterns. Space‐time pattern mining was employed to comprehend the trends according to the diagnosis time and addresses of patients. Finally, the GLR and Multiscale MGWR methods were employed to explain the distribution of TB patients in the city center according to neighborhoods. The results of the Moran's I analysis yielded a Moran's Index of 0.13, a z‐score of 5.28, and a *p*‐value of 0.000, indicating a statistically significant clustering of patients in specific neighborhoods. The LISA results indicate that while the same situation is not observed in the neighborhoods within the historic city center, the peripheral neighborhoods are classified as a High‐High Cluster, which is statistically significant. The Optimized Hot Spot analysis detected significant hot spot areas at the 90% and 95% confidence levels in the southeastern region of the city, while cold spot areas were identified at the 90% confidence level in the northern neighborhoods. The results of the space‐time pattern mining analysis indicate the presence of an upward trend at 13 locations within the city. Furthermore, five new hotspots were identified in the most recent period, whereas no statistically significant hotspots were observed in the preceding period. This finding suggests that there have been regional shifts in the location of TB cases over time. The analyses conducted in Şanlıurfa reveal a concentration of TB cases in specific neighborhoods, including Devteyşti, Eyyüp Nebi, Konuklu, Eyüpkent, and Hayati Harrani.

The GLR and MGWR results indicate that there are six neighborhoods (Eyyüp Nebi, Süleymanşah, Hayati Harrani, Selçuklu, Osmanlı, and Yenice) in the urban areas of Şanlıurfa where the number of TB patients is positively associated with the four variables. It is evident that these areas are predominantly situated in socio‐economically disadvantaged communities. The results of the cold spot analysis indicate that the historical city center and northeastern neighborhoods exhibit a low prevalence of TB cases at a 95% confidence level. This observation suggests the possibility of superior living conditions and more comprehensive health services in these neighborhoods, which collectively contribute to more effective TB control. Furthermore, the MGWR model has been shown to more effectively elucidate the local interrelationships between the prevalence of TB and the variables present within the urban context. This is evidenced by a higher fit value (adjusted *R* ^2^ = 0.75) in comparison to the GLR model. This finding suggests that the MGWR model is particularly adept at identifying local factors that influence the incidence of TB cases. The MGWR model demonstrates that the impact of variables is not consistent across the city, but rather exhibits variation across different neighborhoods.

The Global Burden of Disease study has yielded significant findings regarding the global health impacts of ambient air pollution. In 2013, ambient particulate matter air pollution (PM_2.5_ = particles with an aerodynamic diameter ≤2.5 μm) was estimated to cause 2.9 million attributable deaths (Brauer et al., 2016; Forouzanfar et al., 2016). A substantial body of research has documented the correlation between high levels of ambient air pollutants and TB endemicity in numerous countries. A substantial proportion of the global TB burden, accounting for 43% of cases worldwide, is concentrated in the urban populations of countries such as India, Indonesia, and China, which are disproportionately exposed to elevated levels of outdoor air pollution (Chen et al., 2016; Cohen et al., 2017; Kim, 2014; Popovic et al., 2019). A systematic review of epidemiologic studies conducted in Asia, Europe, and North America revealed that long‐term and short‐term exposure to outdoor air pollutants (PM_2.5_, PM_10_, NO_2_, and SO_2_) is associated with TB culture positivity, TB incidence, TB‐related hospitalization, and TB‐related mortality (Popovic et al., 2019). A subsequent meta‐analysis, which incorporated health records from 1946 to 2022, further substantiated the association between ambient air pollution and lung TB incidence (Dimala & Kadia, 2022). A subsequent meta‐analysis conducted in China found that long‐term exposure to PM_10_, SO_2_, or NO_2_ increased the risk of TB (Xiang et al., 2021). A subsequent study in China demonstrated that TB incidence is spatially clustered and that factors such as temperature, humidity, precipitation, PM_10_, PM_2.5_, O_3_, NO_2_, and SO_2_ have an impact on TB incidence (Li et al., 2022). Sun et al. conducted a study in Beijing, China, that revealed a strong correlation between the occurrence of TB and the increase in air pollutants, including O_3_ and NO_2_ (Sun et al., 2023).

In the present study, a significant positive correlation was identified between the mean PM 2.5 level and the number of TB patients in 10 neighborhoods. The GLR model demonstrated an explanatory power of 52% (Adjusted *R* ^2^ = 0.52) in elucidating the number of TB patients in these areas. The findings indicate that the prevalence of TB is higher in areas characterized by a continuous urban fabric, PM_2.5_ average, and dense transportation infrastructure. The observation of a high concentration of TB cases in neighborhoods with low socio‐economic status suggests that inadequate living conditions and challenges in accessing health services may contribute to the spread of TB. The identification of a statistically significant correlation and spatial clustering between the incidence of acute upper respiratory tract infections and the incidence of TB in specific regions suggests that respiratory tract infections may act as a catalyst for the transmission of TB and that these two health conditions may share common risk factors.

While respiratory diseases are generally believed to be transmitted through close contact, mounting evidence indicates that transmission can occur via multiple avenues, including direct or indirect contact, large droplets, and airborne particles (Gao et al., 2021). It is estimated that nearly all cases of tuberculosis infection occur as a result of airborne transmission of droplets containing live bacteria (dried mucus droplets) from an individual with active TB and sputum acid‐fast bacilli (AFB positive) (Torrelles & Schlesinger, 2017). A systematic review comparing the outcomes of influenza/TB co‐infection with influenza alone concluded that influenza can facilitate the progression of latent TB to active TB disease, alter the clinical presentation of TB, and possibly exacerbate pulmonary TB (Walaza et al., 2020). A comparable scenario pertains to the relationship between TB and SARS‐CoV‐2 infection disease (Covid‐19). Clinical evidence indicates that individuals with active TB disease are predisposed to developing TB infection or having latent TB disease reactivated, and that underlying TB disease can exacerbate the severity of SARS‐CoV‐2 infection (Shariq et al., 2022). A meta‐analysis examining the impact of SARS‐CoV‐2 on co‐infections found that the mortality rate of patients with SARS‐CoV‐2/M. tuberculosis co‐infection was twice as high (Sarkar et al., 2021). A recent study conducted in India revealed that 47.1% of hospitalized patients with confirmed cases of COVD‐19 had evidence of co‐infection (Sreenath et al., 2021). A study conducted in China revealed a higher prevalence of co‐infection in patients with confirmed cases of COVD‐19, with a rate of 94.2% (Lai et al., 2020). The study also demonstrated a statistically significant positive correlation between the total number of acute upper respiratory tract infections in multiple and unspecified regions in urban neighborhoods of Şanlıurfa and the number of TB patients.

The study was subject to some kind of limitations. First of all, it is constrained by the utilization of center points for the determination of patients' locations, owing to the unavailability of reliable data concerning the door numbers of patients' residences. Additionally, the relatively modest sample size of 458 patients may be a contributing factor to the absence of statistical significance in trend and emerging hot spot analyses across the majority of areas. In the GLR and MGWR methods, despite the development of a comprehensive model incorporating approximately 100 independent variables at the neighborhood level from official sources, the model with the highest accuracy (lowest AIC) consisted of only four variables. Despite the incorporation of environmental factors, such as air pollution (PM 2.5), into the analysis, the absence of sufficient detail regarding other potential environmental impacts, including water pollution and housing quality, may limit the analysis's findings. Additionally, the absence of a comprehensive examination of health service accessibility hinders a comprehensive understanding of their impact on TB control, thereby limiting the overall validity of the results. While the findings presented in this study represent a significant advancement in our understanding of the spatial dynamics of tuberculosis, there are certain improvements that merit consideration to elevate the study to a higher level in the future. The first one is related to the diversity of the data. The study's sample size and field diversity can be augmented. Furthermore, individual and environmental factors can be explored in greater depth by analyzing the patients' history (e.g., duration of residence at the specified address, working environment, lifestyle, and other factors that may be relevant) using qualitative and quantitative methods. Beyond environmental factors, such as air pollution, a more extensive data set may encompass water pollution, noise levels, and housing quality. In addition to the GLR and MGWR methods that have been utilized, machine learning and AI‐assisted modeling techniques can be employed. These approaches can be useful in exploring more complex relationships and enhancing predictive power. Furthermore, geographically based nonlinear methods (GeoRandomForest) can be incorporated. The acquisition of additional information about the urban environment where patients reside can be facilitated through Google, Mapillary, or open‐source panoramic street images. These images can then be processed through various image‐processing techniques and integrated into analysis processes. This study examined spatio‐temporal trends over a specific time period. Utilizing extended temporal data sets, the investigation delved into the assessment of seasonal, annual, and climatic variations that exert influence on the dynamics of TB. Subsequent studies may benefit from incorporating community‐based surveys and in‐depth interviews to ascertain the socio‐economic status of TB patients, their access to health services, and their level of awareness. This methodological approach will facilitate a more nuanched understanding of the impact of socio‐cultural factors on TB. It is recommended to further expand multidisciplinary methods that combine new areas in addition to geography, medicine, public health, and environmental sciences to understand TB. Specifically, the integration of health strategies within socio‐economic development initiatives merits consideration. Finally, it would be useful to carry out international comparative studies. A comparison of TB dynamics in Şanlıurfa with those in other regions can facilitate a more nuanced understanding of the interplay between global trends and local factors. Research at regional and national levels can contribute to the formulation of broader health policies. These recommendations are intended to not only further the study of TB in Şanlıurfa but also to advance the field of TB control and spatial epidemiology in general.

## 6 Conclusion

The importance of spatial analyses in the delivery and location of health services is increasing. The analyses conducted in Şanlıurfa have revealed data that may prove instrumental in the distribution of health services and the safeguarding of health workers.

Firstly, an examination of the distribution of patients according to their neighborhoods reveals a high concentration of patients in specific neighborhoods. This underscores the necessity for the consolidation of health services in these locations. However, the observation that this density is lower in some rural neighborhoods highlights the existence of inequities in access. A cluster analysis of the distribution of patients across neighborhoods reveals imbalances in the provision of health services. The concentration of patients in specific neighborhoods underscores the necessity for the reinforcement of the health infrastructure in these locations. The relatively low concentration of patients in rural areas underscores the challenges associated with accessing health services in these settings. Spatial analyses demonstrate that spatial density varies according to the time of diagnosis. Such analyses can serve as a valuable tool for anticipating potential emergencies and allocating resources in a more efficient manner. The planning and delivery of health services must be informed by a careful consideration of spatial analyses. Such analyses can facilitate improvements in public health by ensuring the more equitable and effective delivery of health services.

It is imperative to reinforce health infrastructure in order to enhance the efficacy of TB diagnosis and treatment services. It is imperative that training of health personnel, provision of modern medical equipment, and facilitation of access to medicines be ensured. Furthermore, it is essential to implement follow‐up systems to enhance patient adherence to treatment regimens and expand home care services.

The implementation of these measures to combat TB in Şanlıurfa will serve to prevent the further spread of the disease and to enhance the overall public health of the region. Targeted interventions, particularly in at‐risk neighborhoods, control of air pollution, social and economic support programs, special health services for refugees, and policies and strategies to strengthen general health services will play a pivotal role in controlling TB. The results of this study demonstrate that a comprehensive spatial analysis of TB cases in Şanlıurfa province illuminates the interrelationship between socio‐cultural and environmental factors, indicating that TB is concentrated in specific regions. To effectively reduce and control the spread of TB, it is essential to implement comprehensive health policies and intervention strategies, particularly in high‐risk areas.

## Conflict of Interest

The authors declare no conflicts of interest relevant to this study.

## Data Availability

Among the spatial data used in the study, data with sharing permissions are publicly available at Çelik et al. (2024).

## References

[gh270040-bib-0001] Alene, K. A. , Xu, Z. , Bai, L. , Yi, H. , Tan, Y. , Gray, D. J. , et al. (2021). Spatiotemporal patterns of tuberculosis in Hunan province, China. International Journal of Environmental Research and Public Health, 18(13), 6778. 10.3390/ijerph18136778 34202504 PMC8297355

[gh270040-bib-0002] Anselin, L. (1995). Local indicators of spatial association—LISA. Geographical Analysis, 27(2), 93–115. 10.1111/j.1538-4632.1995.tb00338.x

[gh270040-bib-0003] Barberis, I. N. B. , Galluzzo, L. , & Martini, M. (2017). The history of tuberculosis: From the first historical records to the isolation of Koch's bacillus. The Journal of Preventive Medicine and Hygiene (JPMH). PMC5432783 28515626

[gh270040-bib-0004] Beiranvand, R. , Karimi, A. , Delpisheh, A. , Sayehmiri, K. , Soleimani, S. , & Ghalavandi, S. (2016). Correlation assessment of climate and geographic distribution of tuberculosis using geographical information system (GIS). Iranian Journal of Public Health, 45(1), 86–93. 27057526 PMC4822399

[gh270040-bib-0005] Brauer, M. , Freedman, G. , Frostad, J. , van Donkelaar, A. , Martin, R. V. , Dentener, F. , et al. (2016). Ambient air pollution exposure estimation for the global burden of disease 2013. Environmental Science & Technology, 50(1), 79–88. 10.1021/acs.est.5b03709 26595236

[gh270040-bib-0006] Çelik, M. , Döker, M. F. , Kırlangıçoğlu, C. , Ünsal, Ö. , Gökçeoğlu, S. , Ceylan, M. R. , & Karabay, O. (2024). Sanliurfa tuberculosis [Dataset]. Zenodo. https://zenodo.org/records/13933184

[gh270040-bib-0007] Chen, K. , Chuang, K. , Liu, H. , Lee, K. , Feng, P. , Su, C. , et al. (2016). Particulate matter is associated with sputum culture conversion in patients with culture‐positive tuberculosis. Therapeutics and Clinical Risk Management, 12, 41–46. 10.2147/TCRM.S92927 26792994 PMC4708199

[gh270040-bib-0009] Cohen, A. J. , Brauer, M. , Burnett, R. , Anderson, H. R. , Frostad, J. , Estep, K. , et al. (2017). Estimates and 25 year trends of the global burden of disease attributable to ambient air pollution: An analysis of data from the global burden of diseases study 2015. Lancet, 389(10082), 1907–1918. 10.1016/S0140-6736(17)30505-6 28408086 PMC5439030

[gh270040-bib-0010] Copernicus Land Monitoring Service . (2024). Urban Atlas. Retrieved from https://land.copernicus.eu/en/products/urban‐atlas

[gh270040-bib-0011] Cui, Z. , Lin, D. V. C. , Zhao, J. , Lin, M. , Ou, J. , & Zhao, J. (2019). Spatiotemporal patterns and ecological factors of tuberculosis notification: A spatial panel data analysis in Guangxi, China. PLoS One, 14(5), e0212051. 10.1371/journal.pone.0212051 31048894 PMC6497253

[gh270040-bib-0012] Dangisso, M. H. , Datiko, D. G. , & Lindtjørna, B. (2020). Identifying geographical heterogeneity of pulmonary tuberculosis in southern Ethiopia: A method to identify clustering for targeted interventions. Global Health Action, 13(1). 10.1080/16549716.2020.1785737 PMC7480636 32746745

[gh270040-bib-0013] De Abreu E Silva, M. , Di Lorenzo Oliveira, C. , Teixeira Neto, R. G. , & Camargos, P. A. (2016). Spatial distribution of tuberculosis from 2002 to 2012 in a midsize city in Brazil. BMC Public Health, 16(1), 1–8. 10.1186/s12889-016-3575-y 27581749 PMC5007730

[gh270040-bib-0014] Dimala, C. A. , & Kadia, B. M. (2022). A systematic review and meta‐analysis on the association between ambient air pollution and pulmonary tuberculosis. Scientific Reports, 12(1), 1–13. 10.1038/s41598-022-15443-9 35788679 PMC9253106

[gh270040-bib-0015] ESRI . (2024a). How create space time cube by aggregating points works. ArcGIS Desktop. Retrieved from https://desktop.arcgis.com/en/arcmap/latest/tools/space‐time‐pattern‐mining‐toolbox/learnmorecreatecube.htm#GUID‐FD6C2D31‐FA88‐4460‐8009‐564166ACF391

[gh270040-bib-0016] ESRI . (2024b). How emerging hot spot analysis works. ArcGIS Pro. Retrieved from https://pro.arcgis.com/en/pro‐app/latest/tool‐reference/space‐time‐pattern‐mining/learnmoreemerging.htm

[gh270040-bib-0017] Fatima, I. , Shaikh, W. , Ghafoor, A. , Shaikh, A. , & Abro, S. (2021). The spatial‐temporal epidemiology analysis of tuberculosis disease in Pakistan. Quaid‐e‐Awam University Research Journal of Engineering, Science & Technology, 19(2), 60–67. 10.52584/qrj.1902.10

[gh270040-bib-0018] Fatima, M. , Butt, I. , Firouraghi, N. , Khalil, M. , & Kiani, B. (2024). Space‐time analysis of tuberculosis (2016–2020) in South Punjab, Pakistan. Geojournal, 89(1), 1–13. 10.1007/s10708-024-11020-x

[gh270040-bib-0019] Forouzanfar, M. H. , Afshin, A. , Alexander, L. T. , Biryukov, S. , Brauer, M. , Cercy, K. , et al. (2016). Global, regional, and national comparative risk assessment of 79 behavioural, environmental and occupational, and metabolic risks or clusters of risks, 1990–2015: A systematic analysis for the global burden of disease study 2015. Lancet, 388(10053), 1659–1724. 10.1016/S0140-6736(16)31679-8 27733284 PMC5388856

[gh270040-bib-0020] Fotheringham, A. S. , Yang, W. , & Kang, W. (2017). Multiscale geographically weighted regression (MGWR). Annals of the Association of American Geographers, 107(6), 1247–1265. 10.1080/24694452.2017.1352480

[gh270040-bib-0021] Fotheringham, A. S. , Yue, H. , & Li, Z. (2019). Examining the influences of air quality in China’s cities using multi‐scale geographically weighted regression. Transactions in GIS, 23(6), 1444–1464. 10.1111/tgis.12580

[gh270040-bib-0022] Gao, C. X. , Li, Y. , Wei, J. , Cotton, S. , Hamilton, M. , Wang, L. , & Cowling, B. J. (2021). Multi‐route respiratory infection: When a transmission route may dominate. Science of the Total Environment, 752, 141856. 10.1016/j.scitotenv.2020.141856 32889280 PMC7439990

[gh270040-bib-0023] Getis, A. , & Ord, J. K. (1992). The analysis of spatial association by use of distance statistics. Geographical Analysis, 24(3), 189–206. 10.1111/j.1538-4632.1992.tb00261.x

[gh270040-bib-0024] Goodchild, M. F. (1986). Spatial autocorrelation (pp. 1–56). Geo Books.

[gh270040-bib-0025] Griffith, D. A. (1987). Spatial autocorrelation: A primer. Resource publications in geography. Association of American Geographers. Retrieved from https://books.google.com.tr/books/about/Spatial_autocorrelation.html?id=JYspAQAAMAAJ&redir_esc=y

[gh270040-bib-0026] Guo, C. , Du, Y. , Shen, S. Q. , Lao, X. Q. , Qian, J. , & Ou, C. Q. (2017). Spatiotemporal analysis of tuberculosis incidence and its associated factors in mainland China. Epidemiology and Infection, 145(12), 2510–2519. 10.1017/S0950268817001133 28595668 PMC9148796

[gh270040-bib-0027] Jinou, C. Y. Q. , Yang, R. , Li, L. , Hou, J. , Lu, K. , & Xu, L. (2019). The characteristics of spatial‐temporal distribution and cluster of tuberculosis in Yunnan Province, China, 2005–2018. BMC Public Health, 19(1), 1715. 10.1186/s12889-019-7993-5 31864329 PMC6925503

[gh270040-bib-0028] Kendall, M. G. (1990). Rank correlation methods (5th ed.). Oxford University Press.

[gh270040-bib-0029] Kiani, B. , Rahmati, A. R. , Bergquist, R. , Hashtarkhani, S. , Firouraghi, N. , Bagheri, N. , et al. (2021). Spatio‐temporal epidemiology of the tuberculosis incidence rate in Iran 2008–2018. BMC Public Health, volume, 21. 10.1186/s12889-021-11157-1 PMC8186231 34098917

[gh270040-bib-0030] Kim, J. (2014). Is ambient air pollution another risk factor of tuberculosis? The Korean Journal of Internal Medicine, 29(2), 170–172. 10.3904/kjim.2014.29.2.170 24648798 PMC3956985

[gh270040-bib-0031] Lai, C. C. , Wang, C. Y. , & Hsueh, P. R. (2020). Co‐infections among patients with COVID‐19: The need for combination therapy with non‐anti‐SARS‐CoV‐2 agents? Journal of Microbiology, Immunology, and Infection, 53(4), 505–512. 10.1016/j.jmii.2020.05.013 PMC7245213 32482366

[gh270040-bib-0032] Li, H. , Ge, M. , & Zhang, M. (2022). Spatio‐temporal distribution of tuberculosis and the effects of environmental factors in China. BMC Infectious Diseases, 22(1), 565. 10.1186/s12879-022-07539-4 35733132 PMC9215012

[gh270040-bib-0033] Li, L. , Xi, Y. , & Ren, F. (2016). Spatio‐temporal distribution characteristics and trajectory similarity analysis of tuberculosis in Beijing, China. International Journal of Environmental Research and Public Health, 13(3), 291. 10.3390/ijerph13030291 26959048 PMC4808954

[gh270040-bib-0034] Li, T. , Yang, C. H. , He, J. G. , Li, Y. K. , Xiao, Y. , Li, J. , et al. (2017). Spatial‐temporal distribution of smear positive pulmonary tuberculosis in Liangshan Yi autonomous prefecture, Sichuan province, 2011–2016. Zhonghua Liuxingbingxue Zazhi, 38(11), 1518–1522. 29141341 10.3760/cma.j.issn.0254-6450.2017.11.016

[gh270040-bib-0035] Liao, J. , Qin, Z. , Zuo, Z. , Yu, S. , & Zhang, J. (2016). Spatial‐temporal mapping of hand foot and mouth disease and the long‐term effects associated with climate and socio‐economic variables in Sichuan Province, China from 2009 to 2013. Science of the Total Environment, 563–564, 152–159. 10.1016/j.scitotenv.2016.03.159 27135578

[gh270040-bib-0036] Liu, M. Y. , Li, Q. H. , Zhang, Y. J. , Ma, Y. , Liu, Y. , Feng, W. , et al. (2018). Spatial and temporal clustering analysis of tuberculosis in the mainland of China at the prefecture level, 2005–2015. Infectious Diseases of Poverty, 7(1), 1–10. 10.1186/s40249-018-0490-8 30340513 PMC6195697

[gh270040-bib-0037] Liu, Y. , Li, X. , Wang, W. , Li, Z. , Hou, M. , He, Y. , et al. (2012). Investigation of space‐time clusters and geospatial hot spots for the occurrence of tuberculosis in Beijing. International Journal of Tuberculosis & Lung Disease, 16(4), 486–491. 10.5588/ijtld.11.0255 22325066

[gh270040-bib-0038] Loddenkemper, R. , Lipman, M. , & Zumla, A. (2016). Clinical aspects of adult tuberculosis. Cold Spring Harbor Perspectives in Medicine, 6(1), 1–26. 10.1101/cshperspect.a017848 PMC4691808 25659379

[gh270040-bib-0039] Mabaso, M. L. H. , Vounatsou, P. , Midzi, S. , Da Silva, J. , & Smith, T. (2006). Spatio‐temporal analysis of the role of climate in inter‐annual variation of malaria incidence in Zimbabwe. International Journal of Health Geographics, 5(1), 20. 10.1186/1476-072X-5-20 16700905 PMC1513195

[gh270040-bib-0040] Mann, H. B. (1945). Nonparametric tests against trend. Econometrica, 13(3), 245–259. 10.2307/1907187

[gh270040-bib-0041] Ministry of Health of Türkiye . (2024). 2023 annual activity report. Ministry of Health of Türkiye. Retrieved from https://sgb.saglik.gov.tr

[gh270040-bib-0042] Moran, P. A. P. (1950). Notes on continuous stochastic phenomena. Biometrika, 37(1/2), 17–23. 10.2307/2332142 15420245

[gh270040-bib-0043] Natarajan, A. , Beena, P. M. , Devnikar, A. V. , & Mali, S. (2020). A systemic review on tuberculosis. Indian Journal of Tuberculosis, 67(3), 295–311. 10.1016/j.ijtb.2020.02.005 32825856

[gh270040-bib-0044] Ord, J. K. , & Getis, A. (1995). Local spatial autocorrelation statistics: Distributional issues and an application. Geographical Analysis, 27(4), 286–306. 10.1111/j.1538-4632.1995.tb00912.x

[gh270040-bib-0045] Popovic, I. , Soares Magalhaes, R. J. , Ge, E. , Marks, G. B. , Dong, G. H. , Wei, X. , & Knibbs, L. D. (2019). A systematic literature review and critical appraisal of epidemiological studies on outdoor air pollution and tuberculosis outcomes. Environmental Research, 170, 33–45. 10.1016/j.envres.2018.12.011 30557690

[gh270040-bib-0046] Republic of Türkiye Ministry of General Defence Directorate General For Mapping . (2024). Province and district area measurements. Retrieved from https://www.harita.gov.tr/il‐ve‐ilce‐yuzolcumleri

[gh270040-bib-0047] Republic of Türkiye Ministry of Interior TP of MM . (2024). Distribution of Syrians under temporary protection by top 10 provinces. Retrieved from https://en.goc.gov.tr/temporary‐protection27

[gh270040-bib-0048] Sarkar, S. , Khanna, P. , & Singh, A. K. (2021). Impact of COVID‐19 in patients with concurrent co‐infections: A systematic review and meta‐analyses. Journal of Medical Virology, 93(4), 2385–2395. 10.1002/jmv.26740 33331656

[gh270040-bib-0049] Schmidt, C. W. (2008). Linking TB and the environment: An overlooked mitigation strategy. Environmental Health Perspectives, 116(11), 478–485. 10.1289/ehp.116-a478 PMC2592293 19057686

[gh270040-bib-0050] Senyel Kurkcuoglu, M. A. (2023). Analysis of the energy justice in natural gas distribution with Multiscale Geographically Weighted Regression (MGWR). Energy Reports, 9, 325–337. 10.1016/j.egyr.2022.11.188

[gh270040-bib-0051] Shaikh, M. A. , & Malik, N. A. (2019). Spatial cluster analysis of new and relapsed cases of pulmonary tuberculosis by district: Pakistan 2015. Journal of Ayub Medical College, Abbottabad, 31(2), 293–295. 31094136

[gh270040-bib-0052] Shariq, M. , Sheikh, J. A. , Quadir, N. , Sharma, N. , Hasnain, S. E. , & Ehtesham, N. Z. (2022). COVID‐19 and tuberculosis: The double whammy of respiratory pathogens. European Respiratory Review, 31(164), 1–8. 10.1183/16000617.0264-2021 PMC9488123 35418488

[gh270040-bib-0053] Sisman, S. , & Aydinoglu, A. C. (2022). A modelling approach with geographically weighted regression methods for determining geographic variation and influencing factors in housing price: A case in Istanbul. Land Use Policy, 119, 1–23. 10.1016/j.landusepol.2022.106183

[gh270040-bib-0054] Sreenath, K. , Batra, P. , Vinayaraj, E. V. , Bhatia, R. , SaiKiran, K. , Singh, V. , et al. (2021). Coinfections with other respiratory pathogens among patients with COVID‐19. Microbiology Spectrum, 3(1), 9. 10.1128/spectrum.00163-21 PMC8552727 34287033

[gh270040-bib-0055] Sun, S. , Chang, Q. , He, J. , Wei, X. , Sun, H. , Xu, Y. , et al. (2023). The association between air pollutants, meteorological factors and tuberculosis cases in Beijing, China: A seven‐year time series study. Environmental Research, 216, 114581. 10.1016/j.envres.2022.114581 36244443

[gh270040-bib-0056] Torrelles, J. B. , & Schlesinger, L. S. (2017). Integrating lung physiology, immunology, and tuberculosis. Trends in Microbiology, 25(8), 688–697. 10.1016/j.tim.2017.03.007 28366292 PMC5522344

[gh270040-bib-0057] Turkish Statistical Institute . (2023). Address based population registration system results. Retrieved from https://data.tuik.gov.tr/Bulten/Index?p=Adrese‐Dayali‐Nufus‐Kayit‐Sistemi‐Sonuclari‐2023‐49684

[gh270040-bib-0058] Ugwu, C. I. , Chukwulobelu, U. , Igboekwu, C. , Emodi, N. , Anumba, J. U. , Ugwu, S. C. , et al. (2021). Geo‐spatial mapping of tuberculosis burden in Anambra state, south‐east Nigeria. Journal of Tuberculosis Research, 9(1), 51–62. 10.4236/jtr.2021.91004

[gh270040-bib-0059] Ünsal, Ö. , Lotfata, A. , & Avcı, S. (2023). Exploring the relationships between land surface temperature and its influencing determinants using local spatial modeling. Sustainability, 15(15), 11594. 10.3390/su151511594

[gh270040-bib-0060] Vural, E. , & Şahinalp, M. S. (2022). Examination of the impact of particulate matter‐induced air pollution on urban quality of life: A case study of Şanlıurfa city. Turkish Geographical Review(84), 53–66. 10.17211/tcd.1342144

[gh270040-bib-0061] Walaza, S. , Cohen, C. , Tempia, S. , Moyes, J. , Nguweneza, A. , Madhi, S. A. , et al. (2020). Influenza and tuberculosis co‐infection: A systematic review. Influenza Other Respi Viruses, 14(1), 77–91. 10.1111/irv.12670 PMC6928059 31568678

[gh270040-bib-0062] Wardani, R. , & Wahono, P. (2020). Spatio‐temporal dynamics of tuberculosis clusters in Indonesia. Indian Journal of Community Medicine, 45(1), 43. 10.4103/ijcm.ijcm_182_19 32029983 PMC6985962

[gh270040-bib-0063] WHO . (2024). Global tuberculosis report 2023. WHO press. Retrieved from https://www.who.int/teams/global‐tuberculosis‐programme/tb‐reports/global‐tuberculosis‐report‐2023

[gh270040-bib-0064] Xia, L. , Zhu, S. , Chen, C. , Rao, Z. Y. , Xia, Y. , Wang, D. X. , et al. (2020). Spatio‐temporal analysis of socio‐economic characteristics for pulmonary tuberculosis in Sichuan province of China, 2006–2015. BMC Infectious Diseases, 20(1), 433. 10.1186/s12879-020-05150-z 32571231 PMC7310234

[gh270040-bib-0065] Xiang, K. , Xu, Z. , Hu, Y.‐Q. , He, Y.‐S. , Dan, Y. L. , Wu, Q. , et al. (2021). Association between ambient air pollution and tuberculosis risk: A systematic review and meta‐analysis. Chemosphere, 277, 130342. 10.1016/j.chemosphere.2021.130342 33794431

[gh270040-bib-0066] Yang, G. J. , Vounatsou, P. , Zhou, X. N. , Tanner, M. , & Utzinger, J. (2005). A Bayesian‐based approach for spatio‐temporal modeling of county level prevalence of Schistosoma japonicum infection in Jiangsu province, China. International Journal for Parasitology, 35(2), 155–162. 10.1016/j.ijpara.2004.11.002 15710436

[gh270040-bib-0067] Yang, W. (2014). An extension of geographically weighted regression with flexible bandwidths. University of St Andrews. Retrieved from http://hdl.handle.net/10023/7052

[gh270040-bib-0068] Yates, T. A. , Khan, P. Y. , Knight, G. M. , Taylor, J. G. , McHugh, T. D. , Lipman, M. , et al. (2016). The transmission of Mycobacterium tuberculosis in high burden settings. The Lancet Infectious Diseases, 16(2), 227–238. 10.1016/S1473-3099(15)00499-5 26867464

[gh270040-bib-0069] Yilmaz, M. , & Ulubaş Hamurcu, A. (2022). Relationships between socio‐demographic structure and spatio‐temporal distribution patterns of COVID‐19 cases in Istanbul, Turkey. International Journal of Urban Sciences, 26(4), 557–581. 10.1080/12265934.2022.2063160

